# Deep Learning Optimizes the Diagnostic Performance of Coronary Computed Tomography Angiography in Heavily Calcified Coronary Plaques: A Chinese Multicenter Study

**DOI:** 10.31083/RCM49404

**Published:** 2026-09-08

**Authors:** Chunyan Shi, Chengjin Yu, Bin Zhang, Benjamin Ellison, Zhifan Gao, Zhe Fang, Bin Hu, Jiayin Zhang, Ximing Wang, Longjiang Zhang, Heye Zhang, Lei Xu, Rui Wang

**Affiliations:** ^1^Department of Radiology, Beijing Anzhen Hospital, Capital Medical University, 100029 Beijing, China; ^2^School of Big Data and Statistics, Anhui University, 230601 Hefei, Anhui, China; ^3^School of Computer Science and Technology, Anhui University, 230093 Hefei, Anhui, China; ^4^Division of Cardiovascular Imaging, Department of Radiology and Radiological Science, Medical University of South Carolina, Charleston, SC 29425, USA; ^5^Division of Cardiology, Department of Medicine, Medical University of South Carolina, Charleston, SC 29425, USA; ^6^School of Biomedical Engineering, Sun Yatsen University, 518000 Shenzhen, Guangdong, China; ^7^Department of Cardiology, Lixian Town Community Health Service Center, 101400 Beijing, China; ^8^Department of Cardiology, Beijing Anzhen Hospital, Capital Medical University, 100029 Beijing, China; ^9^Diagnostic and Interventional Radiology, Shanghai Jiao Tong University Affiliated First People’s Hospital, 200080 Shanghai, China; ^10^Department of Radiology, Shandong Provincial Hospital Affiliated to Shandong First Medical University, 200080 Jinan, Shandong, China; ^11^Department of Diagnostic Radiology, Jinling Hospital, The Medical School of Nanjing University, 210002 Nanjing, Jiangsu, China

**Keywords:** coronary artery disease, calcinosis, coronary stenosis, artificial intelligence, deep learning

## Abstract

**Background:**

Accurate evaluation of coronary luminal stenosis using coronary computed tomography angiography (CCTA) remains significantly impaired in the presence of severe coronary artery calcification. Calcification-related blooming artifacts frequently lead to overestimation of stenosis severity and reduced diagnostic specificity, representing a persistent and unresolved challenge in clinical practice. Thus, this study aimed to develop and validate a self-supervised deep learning (DL) algorithm to improve the diagnostic performance of CCTA for assessing luminal stenosis in patients with heavily calcified coronary plaques (Agatston score >300), using quantitative coronary angiography (QCA) as the reference standard.

**Methods:**

This multicenter retrospective study enrolled 786 patients with agatston score (AS) >300 who underwent both CCTA and QCA from four Chinese hospitals. The dataset was randomly divided into a training group (80%, n = 628) for algorithm development and a validation group (20%, n = 158). The diagnostic performance of the DL model and radiologists was compared using sensitivity, specificity, positive predictive value (PPV), and negative predictive value (NPV) for detecting ≥50% diameter stenosis at the lesion, vessel, and patient levels. Reporting time was also evaluated.

**Results:**

In the validation cohort, 476 calcified lesions in 213 vessels from 93 patients were confirmed by QCA to have ≥50% diameter stenosis. The DL model demonstrated significantly higher specificity than radiologists at both the vessel level (52% vs. 30%) and patient level (74% vs. 57%) (all *p* < 0.05). In lesions with severe calcification (cross-sectional calcium arc 270°–360°), DL further improved lesion-level specificity and NPV by 27% and 36%, respectively (all *p* < 0.05). In addition, DL markedly reduced reporting time compared with radiologists (61.9 ± 2.56 s vs. 376.6 ± 127.2 s; *p* < 0.001).

**Conclusion:**

The proposed self-supervised DL model enables rapid and more accurate assessment of luminal stenosis in the presence of heavily calcified plaques, with improved diagnostic specificity and greater workflow efficiency.

## 1. Introduction

In view of its high negative predictive value (NPV), coronary computed tomography angiography (CCTA) has assumed the role of a gatekeeper to avoid unnecessary invasive coronary angiography. The results from the SCOT-Heart Trial 5-year analysis demonstrated that a CCTA-guided approach for evaluating stable chest pain decreased downstream testing and significantly reduced cardiovascular events [1]. Therefore, CCTA is recommended as the initial diagnostic test in symptomatic patients [2].

However, the specificity of CCTA is dramatically reduced in the presence of heavily calcified coronary vessels [3,4], which are the most common reasons for false-positive findings, leading to the overestimation of luminal stenosis. This finding, in turn, prompts unnecessary invasive coronary catheterization (ICA) or myocardial perfusion imaging. Thus, CCTA remains limited in its ability to assess luminal stenosis in the presence of extensive calcified plaques [2]. The diagnostic performance of CCTA is severely compromised in patients with heavy coronary calcification. Blooming artifacts caused by dense calcification frequently lead to overestimation of luminal stenosis, high false-positive rates, and low specificity, especially in cases with an Agatston score >300, which indicates a high coronary calcification burden and is associated with an increased risk of adverse cardiovascular events. Conventional deep learning methods rely heavily on supervised training and often fail to effectively distinguish true luminal narrowing from calcification-related artifacts. These limitations result in persistent diagnostic uncertainty and remain unresolved in current clinical practice. Therefore, a robust and reliable method is urgently needed to address these well-recognized challenges.

Several image post-processing methods [5,6] and subtraction CCTA [7] have been introduced to enhance the specificity of CCTA in the presence of severe calcification. However, these methods require either additional postprocessing or increased radiation doses. Studies have demonstrated that deep learning (DL) - assisted coronary artery calcium scoring has the potential to become a fundamental tool for the risk stratification of patients with suspected acute or chronic coronary artery disease (CAD), thereby aiding clinicians in efficiently defining the cardiovascular risk profiles for each patient [8,9]. The application of DL to CCTA images augments the detection of different types of plaques in coronary arteries (no plaque, noncalcified plaques, mixed plaques, and calcified plaques), as well as the quantification and determination of the anatomical significance of coronary stenoses [10]. These findings indicate that a DL model can improve the integration of CCTA-derived plaque information to enhance risk stratification [11]. However, the potential of the DL model to improve the diagnostic performance of CCTA in the presence of heavy calcifications has not yet been fully explored.

Recent computed tomography (CT)-based artificial intelligence studies, including advanced machine learning and lightweight convolutional neural network frameworks for kidney cancer classification [12,13], have demonstrated that optimized image-feature learning can substantially improve diagnostic accuracy while reducing computational burden. However, these studies mainly address oncologic classification tasks rather than the assessment of heavily calcified coronary stenoses, which requires accurate three-dimensional vascular segmentation and stenosis quantification under severe blooming artifacts. This gap motivated us to develop a self-supervised multi-granularity framework specifically tailored for lumen recovery and assessment of stenosis in heavily calcified CCTA.

Conventional image processing techniques and supervised DL models remain limited in differentiating true luminal narrowing from calcification-related blooming artifacts, particularly in the setting of severe coronary calcification, where complex imaging patterns are not fully captured by labeled training data. To address these limitations, we propose a self-supervised deep learning approach that leverages unlabeled CCTA data to learn robust imaging representations, thereby reducing artifact-related bias and improving discrimination between the calcified plaque and the residual lumen. By minimizing reliance on manual annotation, this framework is designed to enhance diagnostic specificity and NPV in heavily calcified lesions, where both visual assessment and conventional methods are suboptimal. Accordingly, this study aimed to develop and validate a self-supervised multi-granularity mask autoencoder (MGMAE) to improve the diagnostic performance of CCTA for assessing coronary luminal stenosis in patients with severe calcification (Agatston score >300).

The main contributions of this study are as follows:

1. We explicitly target the critical clinical challenge of inaccurate stenosis assessment in heavily calcified coronary arteries using CCTA, where artifact interference and false positives are most prominent.

2. We propose a self-supervised deep learning algorithm that can learn robust imaging features without excessive dependence on manual annotation, thereby improving specificity and negative predictive value in severely calcified plaques.

3. We validate the model in a well-defined cohort with an Agatston score >300, using quantitative coronary angiography (QCA) as the reference standard, demonstrating its clinical utility in a high-calcification population.

4. The proposed approach provides a practical solution to reduce artifact-induced misdiagnosis and complements conventional visual interpretation in challenging calcified lesions.

This paper is organized as follows. Section 2 describes the study population, data acquisition, reference standard, and the detailed framework of the proposed self-supervised DL model. Section 3 presents the experimental results, including diagnostic performance metrics and subgroup analyses. Section 4 discusses the main findings and clinical implications, along with study limitations. Section 5 provides the conclusions.

## 2. Methods

### 2.1 Patient Population

This multicenter, retrospective study was approved by the local institutional review board. A total of 820 consecutive patients who underwent CCTA with an agatston score (AS) >300 [14] and QCA were included in this study. All patients were randomly categorized into a training set (80% of all patients) and a validation set (20% of all patients). The exclusion criteria were an AS <300, prior percutaneous coronary intervention, post-coronary artery bypass grafting or valve replacement, and an interval between the CCTA and ICA of more than 30 days.

### 2.2 CCTA Acquisition and Image Reconstruction

All Calcium scoring and CCTA were performed on a 256-row detector CT scanner (Serial No.: 443308CN2, Revolution CT, GE Healthcare, Milwaukee, WI, USA) and a second-generation dual-source CT scanner (Serial No.: 73787, Somatom Definition Flash; Siemens Healthcare, Erlangen, Germany).

Calcium scoring was performed in all patients using prospective electrocardiogram -triggered acquisition, with a 3 mm section thickness and 120 kV. Each CCTA was chosen individually for every patient, depending on their heart rate (HR) and/or rhythm and body mass index (BMI), with the goal of minimizing radiation exposure. The 256-row detector CT scanning parameters were as follows: z-coverage, 14–16 cm; matrix size, 512 × 512; thickness, 0.625 mm; and gantry rotation time, 280 ms. The second-generation dual-source CT scanning parameters were as follows: detector collimation, 2 × 64 × 0.6 mm; gantry rotation time, 280 ms. The tube potentials used were 100–120 kV in patients with BMI ≥25 kg/m^2^, and 100 kV in those with BMI <25 kg/m^2^. The data acquisition window was set at 70%–80% of the R - R interval when the HR was <70 beats/min, or at 250–350 ms after the R wave when the patient’s HR was fast or irregular. Images were reconstructed from raw data using Iterative Reconstruction in Image Space-3 (IRIS, version VA60A, Somatom Definition Flash, Siemens Healthcare, Erlangen, Germany) and Adaptive Statistical Iterative Reconstruction-Veo (ASiR-V) at a blending level of 50% (ASiR-V 50%, version 19MW38.10, Revolution CT, GE Healthcare, Milwaukee, WI, USA). None of the patients was administered nitroglycerin prior to the scan.

Contrast enhancement was achieved by injecting 50–70 mL of iodinated contrast material (350–370 mg I/mL contrast agent) at a rate of 4.5–5 mL/s through an 18-G antecubital intravenous catheter using a dual-syringe injector, followed by a 30 mL saline flush.

### 2.3 CCTA Image Review

All CCTA images were evaluated separately by two radiologists (R.W. with 15 years of experience in cardiac CT imaging and CH.Y.S. with 7 years of experience in cardiac CT imaging). Coronary segments with a diameter of ≥1.5 mm were included in the analysis, and coronary artery stenosis was quantified using the CAD-Reporting and Data System (CAD-RADS) categorization [15]. Obstructed coronary stenosis was defined as ≥50% luminal stenosis [15,16]. We adopted the >50% stenosis threshold because it is the most widely accepted definition of significant coronary artery disease, ensuring comparability with the prior literature, and it allows for a comprehensive assessment of our DL model’s performance in a clinically representative population to support its future clinical applications. The radiologists were blinded to the results of the ICA. The radiologists were permitted to adjust the window and level settings individually for each study. Calcification was measured as the greatest circumferential extent of calcium and graded according to an adapted cross-sectional arc calcification method [3,17]: 1 = minimal calcification (cross-sectional arc calcium <90°), 2 = mild calcification (cross-sectional arc calcium of 90°–180°), 3 = moderate calcification (cross-sectional arc calcium 180°–270°), and 4 = severe calcification (cross-sectional arc calcium 270°–360°). The radiologists interpreted the original dataset and selected the phases independent of image segmentation. The reference standard of significant coronary stenosis (≥50% diameter stenosis) was determined by the consensus interpretation of two experienced radiologists. Any disagreements were resolved via thorough discussion until a unanimous diagnosis was achieved. Therefore, independent readings were not documented separately, and inter‑reader reliability using Cohen’s kappa was not computed.

### 2.4 Deep Learning Algorithm

#### 2.4.1 Development of the DL Model

Fig. 1 shows the workflow of the DL model. An automated end-to-end network was proposed for coronary artery stenosis segmentation under severe calcification. Model pretraining and coronary artery segmentation were based on CCTA images. In the pretraining stage, an MGMAE was constructed for coronary artery segmentation, which comprised a global mask autoencoder (G-MAE) and a local mask autoencoder (L-MAE). The G-MAE reconstructed masked image patches from visible ones, thus providing a comprehensive representation of the image from a global perspective. Subsequently, the L-MAE reconstructed the local image patches to ensure the diversity of the local regions. In the segmentation stage, a topological loss of function was introduced to maintain the continuity and the inherent topological structure of the vessels by penalizing discontinuous regions. According to the MGMAE segmentation results, the reconstructed three-dimensional (3D) blood vessels were measured. The diameter method was used to calculate the degree of plaque stenosis, and the de-blooming performance of the automatic segmentation algorithm on CCTA was evaluated according to the CAD-RADS category [15]. The detailed steps of model implementation, training, and prediction are provided in **Supplementary Material**. The code can be obtained from https://github.com/zhangbbin/mgmae.

**Fig. 1.**
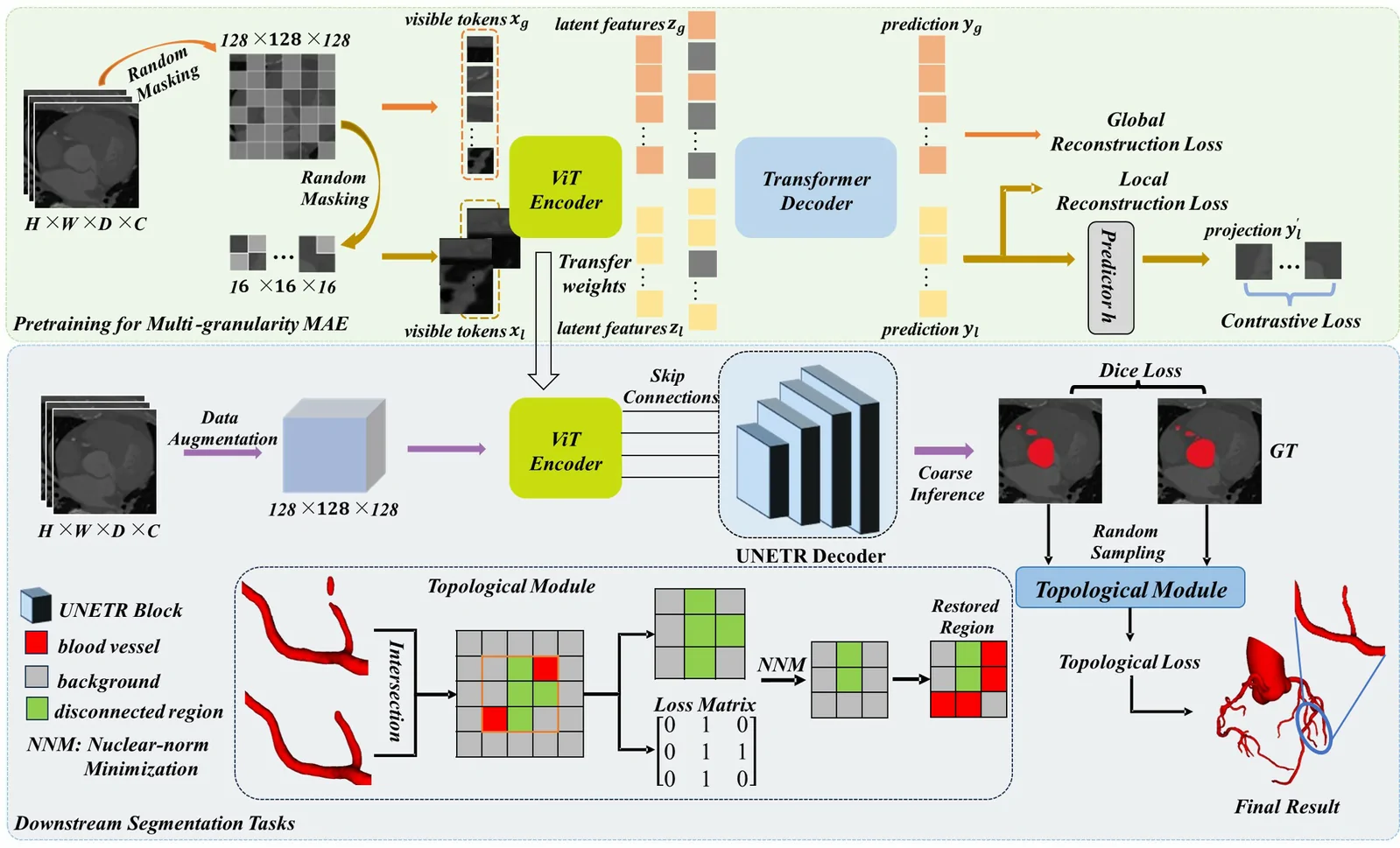
**Framework of MGMAE (Multi-granularity Masked Autoencoder) for Self-Supervised Coronary Artery Segmentation**. The pre-training stage involves G-MAE and L-MAE for image reconstruction tasks and contrastive learning to guide local diversity representation. The fine-tuning stage employs the pre-trained encoder to guide downstream coronary artery segmentation, imposing a topological structure on the segmented label. G-MAE, global mask autoencoder; L-MAE, local mask autoencoder; ViT, vision transformer; UNETR, UNet transformer; GT, ground truth.

To improve reproducibility, the complete workflow of MGMAE is summarized as follows. First, CTA images were resampled to a uniform spacing of 0.4 × 0.4 × 0.4 mm, the HU values were constrained to 300–1000 and normalized by min-max scaling, and the volumes were cropped into 128 × 128 × 128 voxel patches with a 7:3 foreground-background sampling ratio and dynamic augmentation. Second, during self-supervised pretraining, G-MAE reconstructed globally masked patches to learn anatomical context, while L-MAE reconstructed local fine-grained patches to preserve subtle lumen details; contrastive learning was further introduced to enhance the separability between calcification-related and vascular representations. Third, during fine-tuning, the pretrained encoder was transferred to the downstream segmentation task, and Dice loss, weighted cross-entropy loss, and topological loss were jointly optimized to preserve vessel continuity. Finally, three-dimensional coronary trees were reconstructed from the segmentation results, and luminal stenosis was quantified using the diameter method according to the CAD-RADS criteria.

Compared with prior DL-based plaque characterization and CAD-RADS prediction studies, the proposed framework is specifically designed for the assessment of luminal stenosis in heavily calcified plaques. Global MAE preserves the overall vessel course and anatomical context, local MAE focuses on blurred lumen boundaries in calcified segments, contrastive learning increases feature separability among calcification, residual lumen, and adjacent tissues, and topological loss improves continuity of the reconstructed vascular tree for more reliable quantification of diameter-based stenosis.

#### 2.4.2 Experimental Setup

##### 2.4.2.1 Pretraining Phase

Mask ratio: 75%; positional encoding: sinusoidal-cosine encoding; both global and local encoders: 12-layer Transformer architecture with a hidden layer dimension of 768 and 12 multi-head attention heads; optimizer: Adam (learning rate = 1 × 10^–4^, weight decay = 1 × 10^–5^); training epochs: 5000, batch size: 16; loss functions: mean squared error for reconstruction loss, negative cosine similarity for contrastive learning loss; total pretraining loss = reconstruction loss + contrastive learning loss (weight ratio = 1:0.1).

##### 2.4.2.2 Fine-tuning Phase

Decoder: UNet Transformer (UNETR) architecture with 4 decoding modules; optimizer: Adam (learning rate = 1 × 10^–5^, weight decay = 1 × 10^–6^); training epochs: 500, batch size: 4; loss function = Dice loss (α = 0.4) + weighted cross-entropy loss (β = 0.4) + topological loss (γ = 0.2); segmentation evaluation metrics: Dice Similarity Coefficient (DSC), precision, recall, and 95% Hausdorff Distance (HD95).

#### 2.4.3 ICA With Quantitative Analysis

ICA served as the reference standard for stenosis detection and was performed using the conventional Judkins technique. All segments were visually inspected for the presence of stenosis in at least two orthogonal views. QCA was performed using commercially available postprocessing software by two cardiologists (ZF with more than 12 years of experience in coronary catheterization and BH with more than 12 years of experience in coronary catheterization). In this study, the radiologists’ consensus was used as the ground truth for image annotation, model training, and segmentation, whereas QCA was applied as the definitive clinical reference standard for the diagnostic evaluation of ≥50% diameter stenosis. The presence or absence of stenosis ≥50% on QCA was compared with that on CCTA.

### 2.5 Statistical Analysis

Data were analyzed using the GraphPad Prism software (version 8.0; GraphPad Software, San Diego, CA, USA). Independent *t*-tests and χ^2^ tests were used to compare differences between the two groups for continuous and dichotomous variables, respectively. In the present study, both the deep learning model and radiologists provided binary diagnostic outcomes for coronary stenosis, classifying lesions as non-significant stenosis (<50% diameter reduction, coded as 0) or significant stenosis (≥50% diameter stenosis, coded as 1). Since only discrete binary judgments were generated without continuous predictive probabilities or confidence scores, receiver operating characteristic (ROC) curve analysis and area under the curve (AUC) calculations were not feasible. Accordingly, diagnostic performance was evaluated using standard binary classification measures, including sensitivity, specificity, positive predictive value (PPV), NPV, and diagnostic accuracy. Differences in diagnostic performance between the deep learning model and the radiologists were compared using the chi-square test, as appropriate. A two-sided *p*-value < 0.05 was considered statistically significant. The 95% confidence intervals (CIs) for sensitivity, specificity, PPV, NPV, and diagnostic accuracy were calculated using the Wilson/Brown hybrid method for binomial proportions.

## 3. Results

### 3.1 Patient Population

786 patients were enrolled in the study (569 males, 217 females; age, 64 ± 8 years), and 34 patients were excluded because of inadequate quality images. All enrolled patients were randomly categorized into a training set (80% of all patients, n = 628) and a validation set (20% of all patients, n = 158). The demographic data of all 786 patients are presented in Table 1. Statistically significant differences were observed between the training and validation sets in age, BMI, and hyperlipidemia (all *p* < 0.05), while no significant differences were found in other baseline characteristics.

**Table 1.** **Baseline demographics of all patients**.

		All patients	Training set	Validation set		
		(n = 786)	(n = 628)	(n = 158)	t/χ^2^	*p* value
Age		64 ± 8	63 ± 8	65 ± 7	–3.12	0.002
Gender					1.6	0.20
	Male	569 (72%)	461 (73%)	108 (68%)		
	Female	217 (28%)	167 (27%)	50 (32%)		
BMI (kg/m2)		25 ± 5	24 ± 6	25 ± 3	–2.96	0.003
Hypertension		661	526 (80%)	135 (20%)	0.157	0.692
Diabetes		289	233 (81%)	56 (19%)	0.149	0.699
Hyperlipidemia		648	545 (80%)	103 (20%)	39.3	<0.0001
Diseased arteries					2.2	0.332
	One artery	106	79 (75%)	27 (25%)		
	Two arteries	277	224 (81%)	53 (19%)		
	Three arteries	403	325 (81%)	78 (19%)		
Median calcium score		881	912	837	--	--
Tube voltage					0.022	0.882
	100 kV	427	342 (80%)	85 (20%)		
	120 kV	359	286 (80%)	73 (20%)		
Gating technique					0.28	0.871
	Prospective ECG	359	287 (80%)	72 (20%)		
	Retrospective ECG	124	101 (81%)	23 (19%)		
	One-beat	303	240 (79%)	63 (21%)		

BMI, body mass index; ECG, electrocardiogram.

### 3.2 Training Set Performance and Convergence

To further demonstrate the robustness of the proposed model, we additionally analyzed the training-set performance. On the training set, MGMAE achieved a DSC of 92.3% (95% CI: 90.5–94.1%) and an HD95 of 1.28 mm (95% CI: 1.05–1.51 mm) for coronary artery segmentation. For stenosis quantification with QCA as the reference, the model yielded a lesion-level sensitivity/specificity of 89.5%/78.2%, a vessel-level sensitivity/specificity of 91.7%/65.3%, and a patient-level sensitivity/specificity of 93.1%/82.5%. These results were slightly higher than those obtained on the validation set, but no marked performance gap was observed, indicating stable convergence and good generalizability rather than obvious overfitting. The convergence curve and loss trend are provided **in Supplementary Fig. 2 **and **Supplementary Table 1**.

### 3.3 Comparison Between DL and Radiologists Based on Lesion-specific, Per-vessel and Per-Patient Levels

A total of 1090 calcified lesions were found in 632 vessels in the 158 patients on the CCTA in the validation set. A total of 476 calcified lesions in 213 vessels within 93 patients were confirmed with ≥50% diameter stenosis on QCA. The performance of the DL model was better than that of the radiologists at the per-vessel levels as well as per-patient level (Table 2). Diagnostic specificity was significantly improved by using DL than that of the radiologists at the per-vessel level (DL 52% vs. radiologists 30%), per-patient level (DL 74% vs. radiologists 57%) (all *p* < 0.05), respectively.

**Table 2.** **Diagnostic performance of DL and the radiologists on lesion-specific, per-vessel, and per-patient levels**.

Calcium extents		Sen%/Value (95% CI)	Spe%/Value (95% CI)	PPV%/Value (95% CI)	NPV%/Value (95% CI)	LR+	*p* value (McNemar test)
Lesion specific level	Radio. vs. QCA	70 (66–74)	45 (41–49)	50 (46–53)	66 (61–70)	1.3	<0.01
	DL vs. QCA	78 (74–82)	51 (47–55)	55 (52–59)	75 (71–79)	1.6	<0.01
On vessel level	Radio. vs. QCA	81 (75–85)	30 (25–34)	37 (33–41)	75 (68–81)	1.1	0.005
	DL vs. QCA	83 (77–88)	52 (47–57)	47 (42–52)	86 (81–90)	1.7	<0.01
On patient level	Radio. vs. QCA	78 (69–86)	57 (45–68)	72 (63–80)	65 (52–76)	1.8	<0.01
	DL vs. QCA	84 (75–90)	74 (62–83)	82 (73–89)	76 (64–85)	3.2	<0.01

Abbreviations: QCA, Quantitative Coronary Angiography; Sen, sensitivity; Spe, specificity; PPV, positive predictive value; NPV, negative predictive value; LR+, positive likelihood ratio; CI, confidence intervals. *p*-values were calculated using the McNemar test to compare diagnostic performance between each method and the reference standard (QCA).

### 3.4 Comparison Between DL and the Radiologists Based on the Extent of Calcified Plaques

The prevalence of ≥50% diameter stenosis increased with the increasing severity of coronary calcification. At the lesion-specific level, with the application of the DL model, the specificity increased from 32% to 50.0% and from 12% to 39%, while the NPV increased from 51% to 71% and from 43% to 79% in plaques with cross-sectional calcium arcs of 180°–270° and 270°–360°, respectively (Table 3 and Fig. 2A–D). The reporting time for reviewing one case was dramatically reduced from 376.6 ± 127.2 s for radiologists to 61.9 ± 2.56 s for DL (*p* < 0.001) (Fig. 2E).

**Table 3.** **Diagnostic performance of the radiologist and the DL algorithm in different extents of calcification on the lesion-specific level**.

Calcium extents		Sen%/Value (95% CI)	Spe%/Value (95% CI)	PPV%/Value (95% CI)	NPV%/Value (95% CI)	LR+	*p* value (McNemar test)
<90°	Radio. vs. QCA	74 (63–83)	74 (66–81)	58 (47–68)	86 (78–91)	2.9	<0.01
	DL vs. QCA	80 (69–88)	86 (79–90)	73 (61–82)	90 (84–94)	5.6	<0.01
90°–180°	Radio. vs. QCA	52 (42–61)	58 (50–65)	42 (34–51)	67 (59–74)	1.2	0.12
	DL vs. QCA	66 (57–74)	68 (61–75)	56 (47–64)	77 (70–83)	2.1	<0.01
180°–270°	Radio. vs. QCA	64 (57–72)	32 (26–39)	45 (39–52)	51 (42–60)	0.95	0.52
	DL vs. QCA	77 (70–83)	50 (43–57)	57 (50–64)	71 (63–79)	1.5	<0.01
270°–360°	Radio. vs. QCA	87 (81–91)	12 (8–19)	55 (48–61)	43 (28–59)	0.99	0.79
	DL vs. QCA	91 (86–95)	39 (31–47)	65 (58–71)	79 (67–87)	1.5	<0.01

Abbreviations: QCA, Quantitative Coronary Angiography; Sen, sensitivity; Spe, specificity; PPV, positive predictive value; NPV, negative predictive value; LR+, positive likelihood ratio. *p*-values were calculated using the McNemar test to compare diagnostic performance between each method and the reference standard (QCA).

**Fig. 2.**
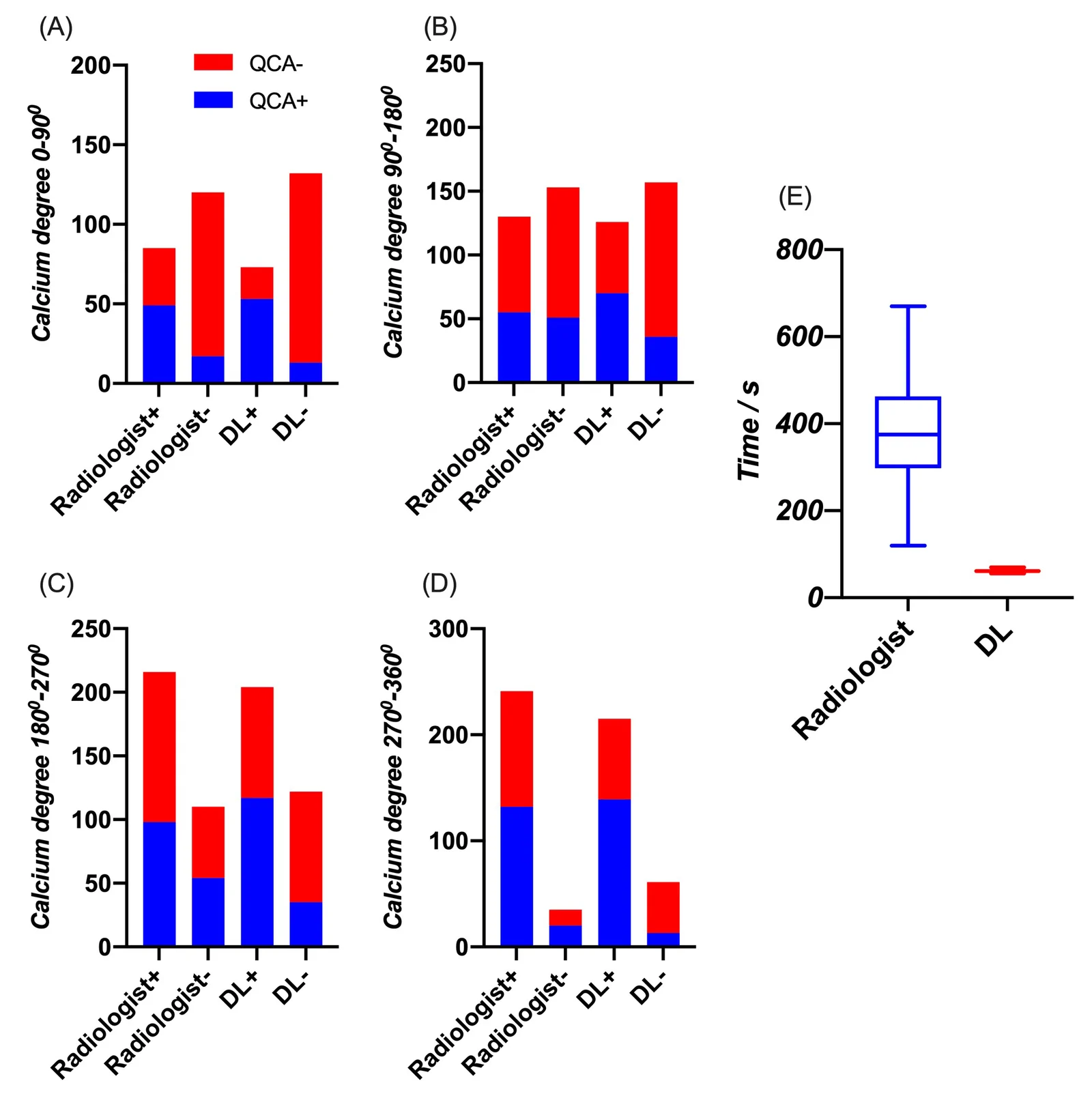
**Diagnostic performance of DL and radiologists on different extents of calcification**. DL, deep learning. (A–D) Distribution of QCA-positive (blue) and QCA-negative (red) lesions stratified by radiologist and DL assessments across increasing cross-sectional calcium arc categories: (A) 0°–90°, (B) 90°–180°, (C) 180°–270°, and (D) 270°–360°. Radiologist+ and Radiologist− denote positive and negative assessments by radiologists; DL+ and DL− denote positive and negative assessments by the deep learning model. (E) Box plot comparing the reporting time per case between radiologists (376.6 ± 127.2 s) and the DL model (61.9 ± 2.56 s) (*p *< 0.001).

The advantage of the DL model became more evident with increasing calcium arc, particularly in the 180°–270° and 270°–360° groups. This pattern is clinically plausible because severe circumferential calcification produces stronger blooming artifacts and greater obscuration of the residual lumen on CCTA, which tends to cause an overestimation of the stenosis by visual assessment. In this study, the radiologist specificity dropped markedly in the severe arc groups, whereas the DL model maintained substantially higher specificity and NPV, suggesting that MGMAE is especially helpful for reducing false-positive assessment of stenosis in heavily calcified lesions. From a clinical perspective, these gains are important because these patients are at greatest risk of unnecessary downstream invasive examinations. The potential effects of lesion location, vessel type, and total calcium burden warrant further investigation in larger cohorts.

### 3.5 Benchmark Against Contemporary AI Methods

To further evaluate the methodological contribution of MGMAE beyond reader comparison, we additionally benchmarked the proposed framework against two contemporary AI methods. UNETR was used as a supervised three-dimensional coronary segmentation baseline trained on the same training/validation split with Dice loss and cross-entropy loss. MAE3D was used as a self-supervised masked autoencoder baseline that adopted conventional single-granularity masked reconstruction without local reconstruction, contrastive learning, or topological constraint. In addition, a commercial calcium de-blooming algorithm (SO-GAN) was included in the stenosis-diagnosis comparison as a clinically available post-processing reference.

MGMAE achieved an 11.3% increase in DSC and a 51.4% decrease in HD95 compared with UNETR, and a 5.2% increase in DSC and a 27.4% decrease in HD95 compared with MAE3D (all *p* < 0.001) (Table 4), indicating that the proposed global-local feature learning and topology-aware optimization improve lumen delineation in heavily calcified segments.

**Table 4.** **Segmentation accuracy comparison between MGMAE and contemporary AI baselines on the validation set**.

Model	DSC (%) (95% CI)	HD95 (mm) (95% CI)	*p* value vs MGMAE
UNETR	78.2 (74.5–81.9)	3.21 (2.85–3.57)	<0.001
MAE3D	84.3 (80.7–87.9)	2.15 (1.82–2.48)	<0.001
MGMAE	89.5 (86.8–92.2)	1.56 (1.23–1.89)	-

Abbreviations: HD95, 95% Hausdorff Distance; DSC, Dice Similarity Coefficient.

The commercial de-blooming algorithm slightly improved sensitivity but did not improve specificity, suggesting limited ability to reduce overestimation of calcification-related stenosis. MAE3D outperformed the commercial algorithm and the radiologist reading, but MGMAE achieved the best overall balance across sensitivity, specificity, NPV, and PPV, supporting the added value of multi-granularity representation learning for heavily calcified coronary assessment (Table 5).

**Table 5.** **Diagnostic performance comparison between MGMAE and alternative methods for assessment of stenosis on the validation set**.

Method	Sensitivity (%)	Specificity (%)	NPV (%)	PPV (%)
Radiologists	70	45	66	50
SO-GAN	73	42	68	49
MAE3D	75	47	71	52
MGMAE	78	51	75	55

## 4. Discussion

This multicenter study helped to validate a self-supervised DL-aided approach (MGMAE) for the evaluation of heavily calcified arteries on CCTA and compared the results with those obtained from QCA. The findings established the superior performance of DL in diagnosing coronary stenosis with heavy calcification, especially in cases of plaques with cross-sectional arc calcium 180°–360°. Moreover, the DL approach significantly improved diagnostic specificity and NPV based on the vessel and patient level, and shortened the reporting time, thereby reducing the potential for unnecessary downstream ICA or myocardial perfusion imaging.

The larger gains observed in the 180°–270° and 270°–360° calcium-arc groups are clinically meaningful because these lesions represent the scenarios in which standard CCTA interpretation is most vulnerable to blooming-related lumen obscuration and assessment of false-positive stenoses. Improved specificity and NPV in these subgroups may therefore reduce unnecessary invasive coronary angiography or additional functional testing.

Luminal stenosis was often overestimated by CCTA in patients with extensive calcified plaques [18]. In this study, calcified plaques were categorized into four types according to an adapted cross-sectional arc. The findings implied that the diagnostic performance of radiologists was significantly reduced in vessels with increased calcium content. This was particularly evident in calcium lesions with a 180°–360° cross-sectional arc of the coronary lumen (the specificity is 12%). Previous methods, such as the de-blooming algorithm [6] and subtraction CCTA [7], have not found widespread clinical application. DL can quantify various parameters of coronary plaque, such as the plaque burden, plaque volume (PV), low-density noncalcified plaques (LD-NCPs), NCPs, calcified plaques (CPs), and lesion length, on CCTA imaging [19,20]. Zreik et al. [10] used a multitask recurrent convolutional neural network in a study on 163 patients for the automatic characterization of plaques. The researchers achieved linearly weighted kappa values of 0.68, 0.66, and 0.67 at the segment, vessel, and patient levels for the binary presence or absence of NCPs, mixed plaques, and CPs, respectively [10]. In this study, DL achieved an accuracy of 0.80 for the detection and characterization of coronary plaques (no plaque, NCPs, mixed plaques, CPs), but in a limited patient volume [10]. This study focused on severe calcified plaques (AS >300) and a relatively larger patient population, with QCA as a reference. Our DL achieved an accuracy of 63% and 80%, and the specificity was improved by 17%–22% for the detection of luminal stenosis on the vessel and patient levels, respectively. Importantly, specificity and NPV were significantly improved by 27%–36% in lesions with 270°–360° calcium using DL compared with the radiologists’ interpretation.

DL effectively enhances the specificity of inexperienced readers and significantly improves the consistency of the diagnosis of coronary stenoses via CCTA [21]. Hence, quantitative plaque assessment using DL-enabled plaque analysis software can provide standardized plaque quantification [22]. The utilization of DL can be considered as a check against diagnostic errors and a tool to reduce physician workload. DL-aided CCTA interpretation determined coronary stenosis and the CAD-RADS category with excellent concordance and expert reader agreement within one CAD-RADS category in 98% of the examinations per-patient and 99.9% of the vessels on a per-vessel basis [23,24]; additionally, it can significantly shorten the reporting time [23]. In our study, the reporting time of the DL algorithm was dramatically reduced compared with the radiologists’ time, from 376.6 ± 127.2 s to 61.9 ± 2.56 s.

Compared with conventional 3D segmentation models, MGMAE enables a self-supervised learning approach to assess both 3D structural and functional features from CCTA images and QCA data. The novel self-supervised MGMAE framework enhances pixel-level differentiation between calcification and blood vessels via local and global MAE. The framework subsequently introduces a topological loss function to preserve the intrinsic topological structure and continuity of the vascular network. The overall diagram is shown in Fig. 1. The pretraining stage includes two main parts: the global mask auto-encoder G-MAE and the local mask auto-encoder L-MAE. The G-MAE captures the overall feature representation of an image by reconstructing occluded image patches. Simultaneously, the MGMAE extracts fine-grained hierarchical image patches from the visible image patches and sends them to L-MAE to reconstruct the local image patches. To ensure the diverse expression of the reconstructed image, the model incorporates a contrastive learning method. The local image patches are classified into different categories based on the Hounsfield unit value, and a contrastive learning loss [25] function is applied to each category of image patches. The intention is to reduce the distance between identical features and increase the diversity of representations of local image patches. During the fine-tuning phase, a pretrained encoder is used to extract the image features, and a UNETR [26] decoder is utilized for coronary artery segmentation. Finally, the model optimizes its parameters to preserve the topological structure of the vascular network by combining Dice loss and topological loss.

Unlike prior DL studies that mainly focused on plaque characterization or CAD-RADS categorization, our framework was designed for the assessment of stenosis in heavily calcified coronary vessels, where accurate lumen delineation is particularly challenging. The present benchmark analysis further shows that the advantage of MGMAE is not limited to comparison with radiologists, but also extends to modern AI baselines. We believe that the complementary combination of G-MAE, L-MAE, contrastive learning, and topological loss provides a task-oriented solution for preserving global vessel context, recovering fine local lumen boundaries, improving feature discrimination, and maintaining anatomically plausible vascular continuity.

This study was based on previous findings and was extended using a novel end-to-end solution that automates calcified plaque analysis. Quantification of calcified plaques is challenging owing to the need to select the best imaging phase, determine the appropriate center line of the coronary artery, and overcome blooming artifacts. Our outcomes demonstrate the feasibility of automatic detection and classification of severely calcified plaques and stenosis using DL. Despite the relatively modest improvement in diagnostic performance conferred by the proposed DL model, particularly for lesions with extensive circumferential calcification, it markedly reduces the time required for CCTA image interpretation, thereby significantly saving time and enhancing clinical workflow efficiency. Such a trade‑off between incremental diagnostic accuracy and substantial time savings represents a meaningful clinical advantage worthy of consideration in routine practice.

Despite the observed improvements in diagnostic performance, lesion-level specificity remained modest (51%), and PPV was limited, indicating that false-positive findings persist even with the assistance of DL. This reflects the inherent complexity of evaluating severely calcified coronary arteries, where blooming artifacts and anatomical overlap continue to limit the accuracy of CCTA. Importantly, the proposed model is not intended to fully overcome these intrinsic limitations but rather to reduce the degree of misinterpretation associated with heavily calcified coronary vessels. Therefore, the DL approach should be considered a complementary tool to support clinical decision-making, improving diagnostic confidence and workflow efficiency.

## 5. Limitations

This study has several limitations. First, only calcified plaques were evaluated, and other plaque components and prognosis were not analyzed. Second, only patients with severe coronary calcification were included, limiting generalizability. Third, data on ischemia-driven revascularization were lacking. Additionally, subgroup analyses were insufficiently powered. Further validation in larger, more diverse cohorts is needed.

## 6. Conclusion

The novel self-supervised deep learning algorithm MGMAE enables accurate quantification of stenosis severity in heavily calcified coronary arteries on CCTA. As a clinical decision support tool, this model has the potential to improve the diagnostic performance of CCTA in patients with heavily calcified plaques.

## Data Availability

The datasets generated and analyzed during the present study will be shared by the corresponding author upon reasonable request.

## Abbreviations

BMI, body mass index; CAD-RADS, Coronary Artery Disease-Reporting and Data System; CAD, coronary artery disease; CCTA, coronary computed tomography angiography; CPs, calcified plaques; DL, deep learning; ICA, invasive coronary angiography; MGMAE, multi-granularity mask autoencoder; NPV, negative predictive value; NCPs, noncalcified plaques; PPV, positive predictive value; QCA, quantitative coronary angiography; ROC, receiver operating characteristic.
