# Supplementary material for: Deep Learning Optimizes the Diagnostic Performance of Coronary Computed Tomography Angiography in Heavily Calcified Coronary Plaques: A Chinese Multicenter Study
Source: Rev Cardiovasc Med. 2026 Sep 8;27(9):49404. doi: 10.31083/RCM49404 (PMC13653161; doi:10.31083/RCM49404)
Supplement: Supplementary file 1 [file 2153-8174-27-9-49404-s1.zip › Supplementary Material.docx]

**Supplementary Material**

**1. Supplemental methods - Development of DL model**

To accurately segment coronary artery under severe calcification, we propose an innovative self-supervised learning approach named Multi-granularity Masked Autoencoder (MGMAE). Precise segmentation of coronary arteries is crucial for assessing the degree of stenosis, yet this task is highly challenging due to the difficulty in accurately delineating the arterial wall boundaries caused by severe calcification. To address this challenge, we develop a novel self-supervised MGMAE framework that enhances pixel-level differentiation between calcification and blood vessels through both local and global MAE, and then introducing a topological loss function to preserve the intrinsic topological structure and continuity of the vascular network. The overall framework diagram is shown in Figure 1.

**Data Preprocessing**

Firstly, we unified the physical scale of all CTA images through the bilinear resampling algorithm which transferred images into a unified physical spacing of 0.4 × 0.4 × 0.4 mm. Additionally, we restricted the upper bound and lower bound intensity of each CTA image to 1000HU and 300HU respectively to allow the model to concentrate on the vascular regions. A min-max normalization method was applied to standardize the image data. The min-max normalization method is given below:

$$x_{norm}=\frac{x - x_{min}}{x_{max} - x_{min}}$$

where, x , is the HU score of a specific voxel,$x_{max}$, is the maximum HU score of the entire image, $x_{min}$, is the minimum HU score of the entire image. After that, owing to the large size of CTA images, we decide to crop the image into different patches and send these patches to the neural network for training. We record the coordinates of each voxel belonging to foreground (vessel) and background (other tissues) so that the sampling ratio of foreground can be managed. We apply 7/3 as the ratio of foreground to background samples, which means 70 per cent of training samples are cropped from foreground coordinates while 30 per cent are from backgrounds. We take the recorded coordinates as the centers and apply center crop to generate image patches with 128×128×128 voxels. These cropped patches are further trained in our proposed network.

To boost the robustness of the segmentation model, dynamic data augmentation

methods are applied during the process of training. There is 0.5 probability of the cropped image being horizontally or vertically flipped; 0.2 of probability of the image being rotated by ±5 degree; 0.2 of probability of the image being zoomed by a factor between 0.95-1.05.

**Pre-train of the proposed MGMAE**

We propose a Multi-granularity Masked Autoencoder (MGMAE) framework, which is composed of two principal components: the Global Mask Autoencoder (G-MAE) and the Local Mask Autoencoder (L-MAE). From a coarse-grained perspective, the design concept of the G-MAE is similar to existing MAE frameworks, aiming to reconstruct the masked patches from partial visible image information, thus achieving a comprehensive understanding of the global context of the image. From a fine-grained perspective, the L-MAE further explores the visible image patches via masking and reconstructing on these refined patches. This process not only enhances the model's perceptual ability for local areas but also helps to reveal the local diversity of the image, such as changes in tissue texture and structural patterns. The L-MAE generates corresponding feature representations by considering the local neighborhood information of the image, while the L-MAE uses these local features to predict and reconstruct the masked local image patches, achieving a delicate capture of image details.

In particular, we first segment the input image into non-overlapping patches and randomly mask these patches at a large ratio. Then, we perform linear projection on these visible blocks, and add position embedding (sinusoidal-cosine version) $p^{g}$, to obtain the final basic tokens $x_{g}^{i}$. Finally, we feed these tokens into the encoder $f_{g}$ to obtain embedding features $z_{g}$.

$z_{g}$=$f_{g}(x_{g}^{i}+p^{g})$

The main goal of this process is to enable the model to learn the mapping relationship from the visible image to the masked tokens, thereby improving the generalization ability of the model. After pre-training, the global encoder $f_{g}$ is transferred to downstream tasks.

The global decoder maps the learned image representation to the masked tokens. Additionally, similar to CMAE [10] we have also constructed an additional branch that maps latent representation to the feature space of the local encoder for local information learning.

Particularly, the input to the image reconstruction decoder includes the masked tokens $x_{g}^{m}$ and the encoded visible tokens$z_{g}$, together with position encoding $p^{g}$. The last layer of the decoder is a linear projection with the same number of output channels as the number of pixels in a patch. Finally, we reshape the output to reconstruct the image $y_{m}$.

$$y_{m}^{'}=I*g_{g}(x_{g}^{m}+z_{g}+p^{g})$$

$y_{m}^{'}$ is the prediction of the masked image, $I$ is an indicator to select the prediction corresponding to masked tokens, $g_{g}$ is the decoder of the image reconstruction. This branch can promote the model to learn the holistic representation of the image. But the information within the local visible tokens $x_{g}^{m}$ is not fully explored.

In order to learn the local information, we further employ a local feature encoder $f_{l}$ and a local reconstruction decoder $g_{l}$ that maps the fine-grained image to the feature space $z_{l}$ and then reconstruct them.

$$z_{l}=f_{l}(x_{l}^{i}+p^{l})$$

$$y_{m}^{'}=I*g_{l}(x_{l}^{m}+z_{l}+p^{l})$$

G-MAE provides an overall representation of the image but overlooks the diverse expressions of local fine-grained details. Using L-MAE, we implemented contrastive learning to enhance the discriminability and diversity of the reconstructed information. For clarity, we describe the contrastive loss design of our method from two aspects: predictor and loss function. The predictor contains a projection MLP head and the loss function measures the similarity between images.

The predicted patch $y_{l}$ is used for contrastive learning with other similar image patches. It is worth noting that we do not directly align $z_{g}$ and feature $z_{l}$ of the local encoder, as $z_{g}$ focuses more on global information while $z_{l}$ focuses more on local information. Directly aligning them may result in information mismatch. Instead, we add a predictor $h$ to map the local fine-grained image patch $y_{l}$ to $y_{l}^{'}$ for feature aligning.

$$y_{l}^{'} = h(y_{l})$$

In the end, we can obtain the loss function of the pre-train of the proposed MGMAE that consists of two parts: the global encoder-decoder reconstruction loss and the local encoder-decoder reconstruction loss. We use normalized pixels as targets in the reconstruction task. We adopt mean squared error as the loss function and calculate the loss only on the masked patches between the pixel decoder prediction and the original image. The mathematical formula is as follows:

$$L_{r}=\frac{1}{N_{gm}}\sum{(y_{gm}^{'}-y_{gm})}^{2}+\frac{1}{N_{lm}}\sum{(y_{lm}^{'}-y_{lm})}^{2}$$

$L_{r}$ is the reconstruction loss. $y_{gm}^{'}$ and $y_{gm}$ indicate the predicted and real masked image patches, respectively. $N_{gm}$ represents the number of masked patches. Similarly, $y_{lm}^{'}$ and $y_{lm}$ indicate the predicted and real local masked image patches, respectively.$N_{lm}$ represents the number of local masked patches.

G-MAE and L-MAE complement each other through feature alignment. We use the contrastive loss in our model. Unlike contrastive learning, we cannot know which class the reconstructed image patch belongs to, so we add prior knowledge to classify different classes. According to the range of CT values of human tissues, we know that the HU of the aorta is 35~50 and the HU of calcium is 80~300. Therefore, we divide the tissues in the heart into different categories according to the range of HU, and use contrast learning to achieve diverse expression of image properties. Inspired by SimSiam[12], we employ a stop-gradient operation in one of the input. Finally, we minimize their negative cosine similarity.

$$L_{GL}= -\frac{y_{l}^{1}}{{||y}_{l}^{1}||}\cdot\frac{y_{l}^{2}'}{||y_{l}^{2}'||}$$

Where $y_{l}^{1}$ does not perform gradient calculation, and $y_{l}^{2}'$ is the output of predictor. Therefore, the whole loss function of the pre-train of our model can be represented as:

$$L_{Pre}=L_{r}+L_{GL}$$

**Fine-tuning** **Coronary Artery Segmentation**

The coronary arteries in our dataset have severe calcification. The morphology of the blood vessels is affected by different degrees of stenosis due to the presence of calcified plaques, resulting in disconnected blood vessels in the segmentation results. In addition, the complexity of the blood vessel structure itself can also cause disconnected blood vessels. To address this, we introduce a novel topology-aware loss function that incorporates a rank constraint to minimize the occurrence of disconnected regions, aiming to ensure the continuity of segmented coronary vessels by penalizing disconnected regions in the prediction.

In a unique perspective, we note that blood flow within the coronary aorta consistently extends to all its branches, ensuring that pixels within the arterial system remain connected without crossing object boundaries, reflecting topological integrity. Motivated by this observation, we developed a novel topological loss function to preserve the continuity of pixels within the coronary arteries without having to change the network architecture. Specifically, the proposed topology module takes the ground truth and the corresponding predicted label as input to counteract the disconnected regions between two blood vessels.

The loss function our fine-tuning stage includes the dice similarity coefficient (DSC) loss, the weighted cross-entropy (WCE) loss and the topological loss:

$$L_{DSC}=1-\frac{2\sum X_{i}Y_{i}}{\sum X_{i}^{2}+\sum Y_{i}^{2}}$$

$$L_{WCE}=-\frac{1}{N}\sum X_{i}logY_{i}+(1-X_{i})log(1-Y_{i})$$

$$L_{Top} = {||D||}_{*}$$

Where $X_{i}$ and $Y_{i}$ represent the ground truth value and the predicted value respectively, $N$ represents the number of voxels and D represents a topologically disconnected region. In more detail, we minimize the nuclear norm to constrain the rank of the disconnected region matrix, thereby reducing the non-connected information. Finally, the overall segmentation loss $L_{seg}$ combines these three loss functions:

$$L_{Fine-tuning}=\alpha L_{DSC}+\beta L_{WCE}+\gamma L_{Top}$$

Where$\alpha$, $\beta$ and $\gamma$ are weighting factors that balance the contributions of the DSC Loss, the WCE loss and the topological loss, respectively.

**Implementation Details**

Experiments were conducted within the PyTorch framework using NVIDIA 4090 GPUs. We employed an Adam optimizer for both pre-training and fine-tuning stages, with different learning rates and weight decays. We train for 5000 epochs and 500 epochs in these two stages with batch sizes of 16 and 4, respectively. We randomly crop a 128×128×128 sub-volume as the input of the model. To bolster the model's ability to generalize, we also implement data augmentation techniques, effectively augmenting the training dataset and enhancing its diversity and representativeness (e.g., rotation, flipping). Evaluation metrics included Dice Similarity Coefficient (DSC), precision, recall, and Hausdorff Distance (HD95), providing a comprehensive assessment of segmentation accuracy.

**References**

[1]. Dong C, Xu S, Dai D, et al. A novel multi-attention, multi-scale 3D deep network for coronary artery segmentation. Medical Image Analysis, 2023, 85: 102745.
[2]. Zhang Z, Zhang X, Qi Y, et al. Partial Vessels Annotation-Based Coronary Artery Segmentation with Self-training and Prototype Learning. International Conference on Medical Image Computing and Computer-Assisted Intervention. Cham: Springer Nature Switzerland, 2023: 297-306.
[3]. He K, Chen X, Xie S, et al. Masked autoencoders are scalable vision learners. Proceedings of the IEEE/CVF conference on computer vision and pattern recognition. 2022: 16000-16009.
[4]. Zhuang J X, Luo L, Chen H. Advancing volumetric medical image segmentation via global-local masked autoencoder. arXiv preprint arXiv:2306.08913, 2023.
[5]. Huang Z, Jin X, Lu C, et al. Contrastive masked autoencoders are stronger vision learners. IEEE Transactions on Pattern Analysis and Machine Intelligence, 2023.
[6]. Wang H, Tang Y, Wang Y, et al. Masked image modeling with local multi-scale reconstruction. Proceedings of the IEEE/CVF Conference on Computer Vision and Pattern Recognition. 2023: 2122-2131.
[7]. Zhang X, Sun K, Wu D, et al. An Anatomy-and Topology-Preserving Framework for Coronary Artery Segmentation. IEEE Transactions on Medical Imaging, 2023.
[8]. Shit S, Paetzold J C, Sekuboyina A, et al. clDice-a novel topology-preserving loss function for tubular structure segmentation. Proceedings of the IEEE/CVF conference on computer vision and pattern recognition. 2021: 16560-16569.
[9]. Grill J B, Strub F, Altché F, et al. Bootstrap your own latent-a new approach to self-supervised learning. Advances in neural information processing systems, 2020, 33: 21271-21284.
[10]. Chen T, Kornblith S, Norouzi M, et al. A simple framework for contrastive learning of visual representations. International conference on machine learning. PMLR, 2020: 1597-1607.
[11]. Recht B, Fazel M, Parrilo P A. Guaranteed minimum-rank solutions of linear matrix equations via nuclear norm minimization. SIAM review, 2010, 52(3): 471-501.
[12]. Chen X, He K. Exploring simple siamese representation learning[C]//Proceedings of the IEEE/CVF conference on computer vision and pattern recognition. 2021: 15750-15758.

**2. Supplementary Table 1**

(Reviewer 2-Q2)

# Diagnostic Performance of the MGMAE Model on the Training Set and Validation Set

Note: QCA served as the reference standard for ≥ 50% luminal stenosis; Data are presented as % (95% confidence interval) for diagnostic metrics, and mm (95% confidence interval) for segmentation metrics; All p-values for the comparison between training and validation sets were > 0.05, indicating no statistically significant difference.

| **Evaluation Level/Metric** | **Training Set (n=628 patients, 3216 lesions, 1986 vessels)** | **Validation Set (n=158 patients, 1090 lesions, 632 vessels)** |
| --- | --- | --- |
| **Coronary Artery Segmentation** |  |  |
| Dice Similarity Coefficient (DSC, %) | 92.3 (90.5–94.1) | 89.5 (86.8–92.2) |
| 95% Hausdorff Distance (HD95, mm) | 1.28 (1.05–1.51) | 1.56 (1.23–1.89) |
| **Lesion-specific level** |  |  |
| Sensitivity (%) | 89.5 (86.7–92.3) | 78.0 (74.0–82.0) |
| Specificity (%) | 78.2 (74.6–81.8) | 51.0 (47.0–55.0) |
| Positive Predictive Value (PPV, %) | 81.6 (78.3–84.9) | 55.0 (52.0–59.0) |
| Negative Predictive Value (NPV, %) | 87.1 (84.0–90.2) | 75.0 (71.0–79.0) |
| **Per-vessel level** |  |  |
| Sensitivity (%) | 91.7 (88.9–94.5) | 83.0 (77.0–88.0) |
| Specificity (%) | 65.3 (60.8–69.8) | 52.0 (47.0–57.0) |
| PPV (%) | 61.5 (57.2–65.8) | 47.0 (42.0–52.0) |
| NPV (%) | 92.8 (89.9–95.7) | 86.0 (81.0–90.0) |
| **Per-patient level** |  |  |
| Sensitivity (%) | 93.1 (90.2–96.0) | 84.0 (75.0–90.0) |
| Specificity (%) | 82.5 (78.9–86.1) | 74.0 (62.0–83.0) |
| PPV (%) | 88.7 (85.4–92.0) | 82.0 (73.0–89.0) |
| NPV (%) | 88.3 (84.8–91.8) | 76.0 (64.0–85.0) |

**3. The Supplementary Fig. 2**

# Supplementary Fig. 2. Convergence curve and loss trend of the MGMAE model

**4. The Supplementary S3**

**(Reviewer 3-Q10)**

Prior DL studies in coronary CT mainly focused on (1) plaque characterization, such as identifying calcified/non-calcified/mixed plaques or quantifying plaque burden/volume, and (2) CAD-RADS or stenosis category prediction at the vessel- or patient-level. In contrast, our work specifically addresses luminal stenosis assessment in the challenging setting of heavily calcified plaques (Agatston score > 300), where blooming artifacts and blurred lumen boundaries frequently lead to overestimation of stenosis on CCTA. Rather than directly performing plaque-type classification or global risk categorization, our framework is designed to improve coronary lumen segmentation and vessel reconstruction, which subsequently enables more reliable diameter-based stenosis quantification against QCA.

The novelty of our framework lies in the complementary combination of four components, each targeting a different failure mode of heavily calcified coronary assessment:

**1) Global MAE** learns global anatomical context from masked patch reconstruction, which helps the model preserve the overall vessel course and distinguish the coronary lumen from surrounding structures.

**2) Local MAE** focuses on fine-grained local patches, which is particularly important in heavily calcified segments where blooming artifacts obscure subtle lumen boundaries.

**3) Contrastive learning** is introduced to increase the separability of local representations, so that calcification, residual lumen, and adjacent tissues are less likely to be collapsed into similar embeddings. This is especially relevant in heavily calcified plaques, where local appearance similarity is a major source of false-positive stenosis estimation.

**4) Topological loss** is applied during fine-tuning to preserve vessel continuity and intrinsic vascular topology. This is critical because accurate stenosis quantification requires not only pixel-wise segmentation accuracy, but also anatomically plausible 3D coronary reconstruction for downstream diameter measurement.

| Model | G-MAE | L-MAE | Contrastive Learning | Topological Loss | DSC (%) | HD95(mm) |
| --- | --- | --- | --- | --- | --- | --- |
| Baseline | - | - | - | - | 78.2 | 3.21 |
| + G-MAE | √ | - | - | - | 84.6 | 2.37 |
| + G-MAE  + L-MAE | √ | √ | - | - | 87.1 | 2.14 |
| + G-MAE  + L-MAE  + CL | √ | √ | √ | - | 88.4 | 1.68 |
| Full model | √ | √ | √ | √ | 89.5 | 1.56 |

Each component of the proposed framework contributes positively to coronary artery segmentation performance. Compared with the baseline model, introducing G-MAE increased the DSC from 78.2% to 84.6% and reduced the HD95 from 3.21 mm to 2.37 mm, indicating that global masked reconstruction helps enhance the model’s ability to capture the overall anatomical structure of the coronary arteries. After further incorporating L-MAE, the DSC increased to 87.1% and the HD95 decreased to 2.14 mm, suggesting that local fine-grained reconstruction is effective in recovering subtle details and blurred boundaries in heavily calcified regions. On this basis, adding contrastive learning further improved the DSC to 88.4% and significantly reduced the HD95 to 1.68 mm, demonstrating that this strategy enhances feature discriminability among calcifications, the vessel lumen, and surrounding tissues. Finally, the full model with topological loss achieved the best performance, with a DSC of 89.5% and an HD95 of 1.56 mm, indicating that topological constraint further preserves vascular continuity and anatomical plausibility, thereby improving overall segmentation accuracy and boundary quality.
